# Autoimmune Encephalitis and Autism Spectrum Disorder

**DOI:** 10.3389/fpsyt.2021.775017

**Published:** 2021-12-17

**Authors:** Paul Whiteley, Ben Marlow, Ritika R. Kapoor, Natasa Blagojevic-Stokic, Regina Sala

**Affiliations:** ^1^ESPA Research, Sunderland, United Kingdom; ^2^Colchester Hospital, East Suffolk and North Essex NHS Foundation Trust, Colchester, United Kingdom; ^3^The Synapse Centre for Neurodevelopment ESNEFT, Colchester, United Kingdom; ^4^Paediatric Endocrinology, Variety Club Children's Hospital, King's College Hospital NHS Foundation Trust, London, United Kingdom; ^5^Faculty of Medicine and Life Sciences, King's College London, London, United Kingdom; ^6^Thinking Autism, London, United Kingdom; ^7^Centre for Psychiatry, Wolfson Institute, Barts and The London School of Medicine and Dentistry Queen Mary University of London, London, United Kingdom

**Keywords:** autism spectrum disorder (ASD), autoimmune encephalitis (AE), autoimmune psychosis (AP), immune system, autoantibodies, neuroinflammation, misdiagnosis

## Abstract

The concept of “acquired autism” refers to the hypothesis that amongst the massive heterogeneity that encompasses autism spectrum disorder (ASD) there may be several phenotypes that are neither syndromic nor innate. Strong and consistent evidence has linked exposure to various pharmacological and infective agents with an elevated risk of a diagnosis of ASD including maternal valproate use, rubella and herpes encephalitis. Autoimmune encephalitis (AE) describes a group of conditions characterised by the body's immune system mounting an attack on healthy brain cells causing brain inflammation. The resultant cognitive, psychiatric and neurological symptoms that follow AE have also included ASD or autism-like traits and states. We review the current literature on AE and ASD. Drawing also on associated literature on autoimmune psychosis (AP) and preliminary evidence of a psychosis-linked subtype of ASD, we conclude that AE may either act as a potentially causative agent for ASD, and/or produce symptoms that could easily be mistaken for or misdiagnosed as autism. Further studies are required to discern the connection between AE and autism. Where autism is accompanied by regression and atypical onset patterns, it may be prudent to investigate whether a differential diagnosis of AE would be more appropriate.

## Introduction

Defined by the core features of social communication issues and the presence of restricted and/or repetitive behaviours that variably but significantly impact on quality of life, autism spectrum disorder (ASD) represents a clinical diagnosis currently based solely on the observation of overt behaviours and developmental history (1). To describe ASD as a heterogeneous label does not do justice to the wide range of presentations that the condition entails. The current system of classification stretches from those who are profoundly disabled by ASD and require round-the-clock care and support to meet their daily needs, to those who are seemingly able to independently navigate the social world with only minimal day-to-day support requirements.

ASD rarely appears in a diagnostic vacuum (2). A multitude of cognitive, behavioural and/or psychiatric conditions are over-represented in ASD (3) many of which can and do significantly impact on quality of life. Diagnoses such as learning disability, attention-deficit hyperactivity disorder (ADHD) and schizophrenia spectrum disorder (SSD) are over-represented in ASD. Issues such as depression, anxiety and various eating disorders can also co-occur with ASD and are commonplace. Various physical health conditions also frequently accompany ASD (4). Most notably, epilepsy and/or seizure disorders and gastrointestinal (GI) disorders (both functional and pathological) can and do affect the expression of ASD, alongside sleeping issues, motor and gait problems and a myriad of other health complications. All these somatic issues have the propensity to affect both the presentation of the core symptoms of ASD and life experiences. For many people diagnosed with ASD, “autism plus” is the rule not the exception (5).

Among the various important debates on the aetiology, presentations, prognosis, and mechanisms underlying ASD (6), the assumptions that (a) ASD is solely an innate condition with universal presentation during the earliest times of development and (b) ASD is a lifelong condition for everyone, continue to be challenged. Various strands of evidence point to differing developmental trajectories in terms of onset patterns and clinical course in ASD. Evidence includes many examples of “acquired autism” where clinically relevant symptoms occur following periods of typical development and subsequent behavioural regression. Similarly, studies of longitudinal ASD presentation show that for some, there is such a significant reduction in symptoms that diagnostic thresholds for ASD are no longer met having previously done so (7).

Historically described as a rare condition, the estimated prevalence of ASD has steadily increased in recent decades, reaching estimates approaching 7% of school-aged children in some regions at the time of writing (8). The reasons for this increase are not yet fully understood. Factors such as increased awareness and diagnostic substitution are probably involved but do not seem to fully account for the sustained increase in numbers of people being diagnosed (9). This increase lends weight to the hypothesis that external (non-genetic) variables may contribute to the onset of ASD.

Various causes of ASD have been identified including syndromic autism (10) (appearing as part of a known genetic condition), ASD accompanying inborn errors of metabolism (11, 12) such as phenylketonuria (PKU) (13), ASD onset following prenatal exposure to specific medications such as sodium valproate (14) and following exposure to infectious diseases such as rubella (15). There have also been many reports of core symptoms and hallmarks of autism emerging as a consequence of viral (16) and bacterial encephalitis (17) in previously developmentally typical children and even adults. There are multiple examples of cases that demonstrate a link between infection and ASD, ranging from developmental and behavioural changes following herpes encephalitis (18, 19) to HIV infection (20–22), where so-called “paediatric NeuroAIDS” patients develop all the core symptoms of ASD as well as numerous accompanying behaviours and presentations (23).

It is instructive to consider the links between autism and infection in light of multiple reports suggestive of a genetic (24, 25) and non-genetic (26) propensity toward issues with immune function in cases of ASD. Despite the heterogeneity of the ASD label, there is evidence for both under- and over-activity of the immune system in ASD. This is exemplified by research reporting abnormalities in immune behaviour in relation to allergy and mast cell functions (27) and inflammatory processes (28) in ASD. Inflammation, and the many and varied inflammatory responses, are the focus of continuing scientific discussion and debate (29, 30) reflective of multiple lines of evidence indicating an important connection. Other literature also highlights the increased risk of autism in the offspring of mothers with poorly controlled immune-related issues in pregnancy (31). An over-exaggerated response to an immune trigger seemingly mirrors the complexity of sensory hypersensitivities and dysautonomia seen in ASD including an increased physiological response to stress (tachycardia, hypertension and raised cortisol/adrenaline levels) (32). Such a physiological response demands much of the metabolic reserve. The “Warburg effect” (aerobic glycolysis) (33) may be relevant, detailing how an exaggerated immune trigger switches oxidative phosphorylation to glycolysis in the race to meet the metabolic demands of an “over-primed” immune system.

Sustained, over-responsive immune signalling-a risk factor for autoimmunity where self is not recognised as self by the immune system, and results in an immune response to the body's own tissues-is also strongly implicated in ASD (34, 35). Findings range from the over-representation of a family history of autoimmune issues as a recognised risk factor for ASD (36), to exposure and reaction to maternal autoantibodies and maternal immune activation during critical periods of gestation (37, 38). Some authors have even gone as far as to talk about “*an immune subtype of the autism spectrum*” (39) as being a specific type of autoimmune disorder.

Consolidating many of the immune system findings in ASD, the specific notion of neuroinflammation as a feature of at least some ASD (40) holds particular importance given the nature of encephalitis, characterised by inflammation of the brain or surrounding tissue. The related use of immune modulating agents in ASD is also gaining recognition (41, 42). Primary immunomodulatory agents such as intravenous immunoglobulin (IVIG) (43), corticosteroids (44, 45), adrenocorticotropic hormone (ACTH) (46) and celecoxib (47) as possible treatments for core autism symptoms have garnered research attention, alongside recognition that other medications widely used in ASD, such as the atypical antipsychotic risperidone for example, have immunomodulatory actions (48).

For all the reasons previously outlined, our first aim is to review the current literature on AE and ASD. Secondly, we review the literature on autoimmune psychosis (AP) and the preliminary evidence of a psychosis-linked subtype of ASD.

## Autoimmune Encephalitis

### Types and Symptoms

Encephalitis is a severe inflammatory neurological disorder that affects the brain. Encephalitis occurs for two reasons: (a) infection attacking the brain, or (b) a person's own immune system attacking the brain in error. These occurrences are known respectively as infectious encephalitis (where encephalitis is predominantly caused by exposure to one or more viral agents) and autoimmune encephalitis (where various facets of the immune system wrongly attack brain tissue akin to an autoimmune reaction or disease).

Autoimmune encephalitis (AE) (49) covers a group of conditions, the most common being Acute Demyelinating Encephalomyelitis (ADEM), LGI1/CASPR2-antibody encephalitis and anti-N-methyl-D-aspartate (anti-NMDA) receptor encephalitis. All types of AE are classed as rare with an estimated incidence of 5–10 per 100,000 people per year. Onset is typically acute and sudden, characterised by a prodromal phase, with symptoms appearing after an illness that includes fever, malaise and headache which mirrors something like influenza. Various symptoms follow affecting behaviour, cognition and neurology, usually within 3 months. Symptoms include seizures, hallucinations and associated symptoms affecting responsiveness and consciousness representative of an altered mental state, communication and memory issues, sleeping issues and the presence of abnormal movements (including dystonia and akinesia) which may or may not be repetitive and/or stereotypical in nature. Following appropriate diagnosis and instigation of a treatment regime, many people make a good recovery from AE, albeit with some heightened risk for relapse and, for some, sustained symptoms in the longer term.

### Identification and Diagnosis

The rarity of AE and limitations to the knowledge base in relation to its symptoms, presentations and pathophysiology create significant hurdles to diagnosis. Discrete types of AE are indistinguishable from each other on the basis of observable symptoms. Further, the constellation of symptoms associated with AE are also typically seen in various other diagnoses covering neurological (seizure disorders, various encephalopathies), psychiatric (psychosis, schizophrenia, drug toxicity) and medical issues (Wernicke encephalitis, neuroleptic malignant syndrome, lymphoma, other neoplastic diseases, mitochondrial diseases). Diagnosis is therefore partially formed on the basis of exclusion of other similarly presenting conditions and also potentially, where interventions for said similarly presenting conditions fail to provide relief from symptoms (or even in some cases, aggravate symptoms).

Given the autoimmune focus in AE, an important diagnostic role is reserved for autoantibody testing for the condition. Various autoantibodies are helpful in diagnosing different types of AE, a full explanation of which is beyond the scope of this paper. Suffice to say that autoantibodies to cell surface proteins and/or intracellular synaptic proteins and receptors can provide important information on the specific nature of AE and guide treatment options. When coupled with other diagnostic measures such as electroencephalogram (EEG), magnetic resonance imaging (MRI) and/or preferential cancer screening, an important clinical picture can be obtained to complement the overt features. That being said, there is also a growing appreciation that AE can potentially manifest alongside negative results following autoantibody testing (50). Various reports have detailed cases of AE accompanied by seronegativity for autoantibodies, to the extent that some clinicians recommend initiating immunotherapy in “criteria-positive cases” but in the absence of autoantibodies.

### Presentation in Children vs. Adults

Despite commonalities in the behavioural, psychiatric and neurological presentation of AE across the age ranges, differences in presentation have been noted according to age and developmental stage. Titulaer and colleagues (51), looking at the records of 500 patients with AE, noted that those under 12 years of age tended to show more pronounced behavioural, seizure and movement disorders compared with older cohorts. Adults, by contrast, tended to show more cognition and memory problems. For children, AE appears to more frequently manifest as agitation, temper tantrums and aggression, often coinciding with speech and language issues such as mutism and echolalia (52), sleep disorder, sensory issues and gait and movement disturbances (53, 54). de Bruijn et al. (55) also discussed the various mimics of AE presentation in children. They noted that possible alternative clinical labels need to be considered when diagnosing paediatric AE, including Gilles de la Tourette syndrome and paediatric autoimmune neuropsychiatric disorders associated with *Streptococcus* infections (PANDAS) whose symptoms may overlap with AE. Many of the symptoms which define AE are also present in ASD. As detailed in the following sections of this paper, there is strong evidence that, particularly in cases of regressive ASD, misdiagnosis of ASD could be at play. A recent prospective national cohort study also observed that SARS-CoV-2 is associated with higher incidence of neurological manifestations and sequelae in hospitalised children and adolescents, in particular those with paediatric inflammatory multisystem syndrome who needed more intensive care. These findings could be explained by a potential mediation from the systemic inflammation through cytokine storm (56).

Another important difference separating paediatric and adult presentation of AE is the elevated presence of tumours in adults. Ovarian teratoma (57) represents one of several malignancies (58) associated with adult female AE. Paediatric AE by contrast, is much less likely to be associated with the presence of this and other germ cell tumours.

The typical profile of autoimmunity associated with AE is also likely to differ according to age. It is important to realise that AE can be diagnosed in children and adults even if all the antibody tests are negative, provided some other criteria are met. The diagnostic algorithm for paediatric AE produced by Cellucci and colleagues (59) accommodates such a presentation.

### Intervention

The essential immune system component to AE means intervention is predominantly aimed at the immune system (60). First line treatment using combinations of steroids, IVIG and plasma exchange (plasmapheresis) are indicated. Secondary use of monoclonal antibodies such as rituximab and the antineoplastic cyclophosphamide may also be indicated in some cases non-responsive to first line interventions. Other clinical options may be used where first and second line treatments do not provide suitable relief from symptoms. Prompt treatment is associated with a more favourable clinical outcome. Importantly too, the recommended treatment options for AE do not differ for children and adults (54).

## Autoimmune Encephalitis and ASD

Despite sound scientific evidence that various immune system irregularities are a feature of ASD and that early-life infection exposure to infections and inflammatory mediators can cause ASD or ASD-like features, or contribute to the severity of symptoms and presentations, there remains some hesitancy toward viewing ASD as a biological condition. Although past psychological explanations in relation to ASD have been roundly dismissed and all-encompassing psychological models on the nature of ASD are in retreat, entrenched views about the label persist. None more so than the assumptions that (a) ASD is solely and universally inborn and innate, and (b) that a diagnosis is immutable and lifelong (7). Such viewpoints are flawed on the basis of various factors, not least the enormous heterogeneity that ASD encompasses, and the emerging data on regression, acquired ASD as well as a significant minority of ASD cases where diagnostic symptom thresholds are no longer met. Such observations are also pertinent when exploring the overlap between ASD and AE.

### Regression in AE Mimics Autistic Regression

The phenomenon of “autistic regression,” denoting a halt in acquisition and/or reversal of previously acquired skills and onset of autistic characteristics, is well documented. Various studies talk about regression as being a significant factor in symptom onset in ASD. Some authors have even gone so far as to suggest that regression in autism “*may be the rule rather than the exception*” (61). Regression in previously acquired skills and developmental milestones is also a facet of AE. Analyses of AE onset patterns, particularly in children, show that various behaviours are associated with the condition, including those that constitute core symptoms used to diagnose idiopathic autism. The behaviours include loss of speech, echolalia, changes in speech patterns or (in)ability to form sounds, loss of eye contact, diminished interest in surroundings, loss of social interaction and lack of interest in age-appropriate play (52).

The rapidity with which these and other symptoms of AE appear (often within weeks) is notable. Dale (62) emphasised this regression in his commentary on the case report by Hacohen et al. (63) describing two children who presented with autistic regression and were subsequently diagnosed with anti-N-methyl-D-aspartate (anti-NMDA) receptor encephalitis. The key issues of “*loss of reactivity to the environment, loss of social reciprocity, and loss of language*” fit precisely with the core symptoms of ASD. Forensic examination of the movement issues that also characterised the case reports described by Hacohen et al., further noted their “stereotypical” dimension, distinct from being extrapyramidal (most commonly associated with antipsychotic medication use). Other independent data describing anti-NMDA receptor encephalitis leading to a diagnosis of autism similarly describe autistic regression as a significant feature (64–66). These and various other reports clearly show that regression accompanying various cases of paediatric AE in particular, can be autistic in nature.

### Further Symptom Overlap

Alongside manifesting the core social communication issues that define ASD, various other behavioural and somatic manifestations that are commonly associated with ASD are also seen in AE. Changes in behaviour and mood, especially the expression of anxiety, irritability and agitation are common in paediatric cases of AE (67). All of these behaviours are also frequently present in ASD, including following autistic regression (68). Such overlap can provide a diagnostic challenge (69) especially during early infancy when ASD typically manifests.

Sleeping problems represent another area of overlap, present in between 40 and 80% of children and adults with ASD (70). Reports of sleep complaints accompanying over 70% of cases of AE (71) have been observed, manifesting as rapid eye movement sleep behaviour disorder, hypersomnia, fragmented sleep and sleep-disordered breathing.

Epilepsy and/or seizure disorders are substantially more prevalent in individuals with ASD compared to the general population. Depending on the definition of epilepsy used and the presence of accompanying learning disability alongside ASD, conservatively at least 10% of people with ASD will also present with epilepsy over a lifetime (72) with a higher proportion at risk of seizures. Likewise, there are elevated levels of ASD occurring in cases of epilepsy (73) following suggestions that epilepsy may lead to ASD (74). The overlap with AE is notable (75) as issues such as autoimmunity and inflammation show potentially important connections to epileptogenesis in AE. An increasing appreciation of the concept of autoimmune epilepsy (76) also maps on to the neurological antibodies noted as causative agents in AE, alongside the notion of refractory epilepsy (not controlled by typical intervention).

We have previously touched upon the issue of movement disorder as being a clinical symptom of AE (62, 63). Florance et al. (67) reporting on the clinical features of paediatric AE, extensively discussed how various motor disorders were present in their cohort. Dyskinesia (involuntary movements) were present in many of their cases, as were behaviours such as odd rigidity and bizarre posturing. The presence of motor issues accompanying ASD has an equally long history; even the first formal descriptions of ASD described gait, odd and rigid posturing and gross motor performance difficulties as part and parcel of symptom presentation (77).

Various other behaviours are commonly observed in both ASD and AE. Issues such as cognitive impairment, feeding issues exemplified by decreased appetite and dysphagia, self-harming behaviours, headaches and continence issues have all been frequently noted in cases of regressive ASD and AE (78). Sensory processing issues, a hallmark of ASD (79) are also present in some cases of AE (49).

Of additional interest, are the reports of febrile illness accompanying regression in ASD (80) which may very well map on to the prodromal period noted in AE onset. Scott et al. (80) observed a stark comparison between those with regressive ASD vs. non-regressive ASD in the rates of febrile illness (30 vs. 0%). “Flu-like” illness is a consistent feature of AE onset. Both accounts point to environmental triggers of immune activation suggesting that inflammatory pathways may be a consistent feature in developmental regression.

### Recovery of Autistic Features

The seminal commentary by Ozonoff (81) talking about “*recovery from autism spectrum disorder*” in the context of various reports of clearly documented ASD in early childhood no longer meeting the criteria for ASD (82, 83) is pertinent to AE. Not least because, following diagnosis of AE and instigation of an appropriate immunotherapy treatment regime, reports of improvements and even resolution of autistic symptoms have been noted. Such reports are at odds with the prevailing “lifelong” narrative that accompanies a diagnosis of ASD despite the heterogeneity that the label entails.

## Autoimmune Psychosis vs. Autoimmune Encephalitis

Complicating the clinical picture on the overlap between AE and ASD is an additional area with the potential for further misdiagnosis: autoimmune psychosis (AP) vs. AE. AP (84) represents a relatively new label characterised by the presence of psychotic behaviours (hallucinations, delusions, incoherent thinking, disorganised behaviour) coupled with positive immunological findings for neuronal antibodies of the type used to diagnose types of AE. Screening and intervention options also mimic those used in AE as opposed to more traditional antipsychotic pharmacotherapy. Whether AP is entirely distinguishable from AE remains a source of debate. For the purpose of this article we accept the separative model as valid based on the currently available evidence. As with the potential for misdiagnosis of ASD in cases of AE and issues around related conditions such as autoimmune epilepsy, there is a requirement for careful screening for AP in cases of sudden onset psychosis (84).

Various intersecting areas unite AE, AP and ASD. Historically, ASD and schizophrenia, a condition that can manifest as psychosis, share important history. Evans (85) detailed the various revisions leading to our modern description of ASD including the important description provided by Creak and colleagues (86) and their nine key features of “*schizophrenic syndrome in childhood*.” These features were eventually condensed into what we now know as ASD, with descriptions such as “abnormal perceptual experience” previously thought to mean hallucinations, morphing into the neurocognitive sensory domain that is widely discussed in ASD today. The partition of schizophrenia and/or psychosis-linked conditions from ASD, whilst complete in diagnostic terms, is however not completely severed. Various studies have observed elevated rates of both ASD and ASD-associated traits in psychosis (87), while others have reported on the late diagnosis of ASD only after the incidence of psychotic events (88). The rates of comorbid ASD in schizophrenia are also elevated (89). As part of an increasingly vocal dialogue regarding the pluralisation of ASD (90), observations of “*a specific subtype of ASD linked to comorbid psychosis*” (91) provide important information about the “cross-reactivity” of ASD and psychosis or psychosis-linked diagnoses in clinical presentation.

At the genetic and biological level, there also remain some important similarities between ASD and psychosis-linked diagnoses. Aside from shared neurological findings, the increasing focus on immune function witnessed in relation to ASD is also mirrored in psychosis and schizophrenia spectrum disorders (SSD) (92). Inflammation and the notion of autoimmunity are key findings in psychosis and SSD, alongside discussions on the usefulness of anti-inflammatory strategies as treatment options. The rise of immunopsychiatry continues at pace. Collectively, such findings suggest commonalities between ASD, AP and AE.

## Where Next?

Although still an area under investigation, there is sound, evidence-based backing for expanding screening for AE where a previously healthy child or adult experiences a developmental regression resulting in the presentation of ASD or ASD-associated features (59, 93). There is adequate evidence that neuroinflammation and encephalitis are relevant to ASD (94) and that the presentation of AE mirrors the presentation of regressive ASD. Only with appropriate screening, can a diagnosis of AE be considered and appropriate intervention put in place. There are various other examples of such forensic examination in action when ASD presents, particularly where regression of previously acquired skills is a feature (95, 96).

Screening for AE in cases manifesting regressive ASD needs to take account of the possibility that those with autistic regression may form a distinct subgroup on the autism spectrum, akin to a predisposition to autoimmune and “inflammatory activation” (80). Furthermore, clinicians need to consider that a diagnosis of ASD is in no way protective of developing AE, which may also very well worsen pre-existing features of ASD (97). Cumulatively, these and other findings offer important clues about how age and developmental maturity are important to the presentation of AE. Combined with other age-dependent factors such as the increased likelihood of AE being due to an underlying tumour in adults compared to an absence of tumours in paediatric AE, the requirement for forensic and wide-ranging examinations when AE is suspected in children or adults is paramount.

Methods for the analysis of autoantibodies and the subsequent use of immune therapies in cases of suspected or confirmed AE manifesting ASD also require further scientific examination. There is currently no guidance on the routine inspection of autoantibodies more generally in cases where ASD is diagnosed; something which complicates calls for preferential screening for AE related autoantibodies in cases of regressive ASD. Further longitudinal investigation of immune therapy for ASD manifesting AE is also required. Inspection of other related factors such as shared genetic variables overlapping between AE and ASD may also provide important information about who may be most susceptible to AE manifesting ASD and new ways of intervening. Overlapping research on the human leukocyte antigen (HLA) system would be an obvious starting point given markers of possible overlap (98, 99).

## Conclusion

There is emerging evidence of a connection between AE and ASD. Multiple reports have described the presentation of ASD and/or autistic features in cases of diagnosed AE. Alongside other important observations of immunological issues being over-represented in ASD and various other types of infection associated with the onset of symptoms, there are important reasons for AE to be treated as a clinical priority in the context of ASD. It can no longer be assumed that regression, for example, is just “a part of autism” without any need for investigations. The cases of ASD appearing alongside AE provide a template for further clinical examination of such regressive onset patterns. There are significant challenges in relation to the assessment and diagnostic pathways toward recognising AE in ASD. Similar challenges are also present with regards to the treatment of AE in this context and in determining whether ASD appearing alongside AE represents a distinct phenotype of ASD or merely a differential diagnosis of ASD.

## Author Contributions

All authors listed have made a substantial, direct, and intellectual contribution to the work and approved it for publication.

## Funding

ESPA Research has received funds from the Robert Luff Foundation, some of which contributed to pay for PW time in the preparation of this manuscript. The Foundation played no role in deciding the content or in the production of the final manuscript.

## Conflict of Interest

RS has been an advisor to and has received honoraria from Takeda. The remaining authors declare that the research was conducted in the absence of any commercial or financial relationships that could be construed as a potential conflict of interest.

## Publisher's Note

All claims expressed in this article are solely those of the authors and do not necessarily represent those of their affiliated organizations, or those of the publisher, the editors and the reviewers. Any product that may be evaluated in this article, or claim that may be made by its manufacturer, is not guaranteed or endorsed by the publisher.
